# An analytically tractable, age-structured model of the impact of vector control on mosquito-transmitted infections

**DOI:** 10.1371/journal.pcbi.1011440

**Published:** 2024-03-14

**Authors:** Emma L. Davis, T. Déirdre Hollingsworth, Matt J. Keeling

**Affiliations:** 1 Mathematics Institute, University of Warwick, Coventry, United Kingdom; 2 Zeeman Institute for Systems Biology and Infectious Disease Epidemiology, University of Warwick, Coventry, United Kingdom; 3 Big Data Institute, University of Oxford, Oxford, United Kingdom; University of California San Diego Division of Biological Sciences, UNITED STATES

## Abstract

Vector control is a vital tool utilised by malaria control and elimination programmes worldwide, and as such it is important that we can accurately quantify the expected public health impact of these methods. There are very few previous models that consider vector-control-induced changes in the age-structure of the vector population and the resulting impact on transmission. We analytically derive the steady-state solution of a novel age-structured deterministic compartmental model describing the mosquito feeding cycle, with mosquito age represented discretely by parity—the number of cycles (or successful bloodmeals) completed. Our key model output comprises an explicit, analytically tractable solution that can be used to directly quantify key transmission statistics, such as the effective reproductive ratio under control, *R*_*c*_, and investigate the age-structured impact of vector control. Application of this model reinforces current knowledge that adult-acting interventions, such as indoor residual spraying of insecticides (IRS) or long-lasting insecticidal nets (LLINs), can be highly effective at reducing transmission, due to the dual effects of repelling and killing mosquitoes. We also demonstrate how larval measures can be implemented in addition to adult-acting measures to reduce *R*_*c*_ and mitigate the impact of waning insecticidal efficacy, as well as how mid-ranges of LLIN coverage are likely to experience the largest effect of reduced net integrity on transmission. We conclude that whilst well-maintained adult-acting vector control measures are substantially more effective than larval-based interventions, incorporating larval control in existing LLIN or IRS programmes could substantially reduce transmission and help mitigate any waning effects of adult-acting measures.

## Author summary

Parity is a term used to describe the number of feeding and egg-laying cycles (or gonotrophic cycles) a mosquito has completed. The parity of a mosquito is an important factor when considering vector-borne infectious diseases, since it takes at least two bloodmeals for transmission to occur—one for the mosquito to pick up infection, and one to pass on infection to a new host. In practice, the more bloodmeals a mosquito has taken, or the higher its parity, the more likely it is to contribute to transmission. Therefore, a population consisting of older mosquitoes will represent a higher risk of transmission. Vector control methods act to reduce the mosquito population and include adult-acting measures (such as long-lasting insecticide-treated nets and indoor residual spraying) and larval-acting measures (such as insecticidal treatment of larval sites). Although these measures all act to reduce population size, they also have different impacts on the age-structure of the population. Here we develop an analytically tractable, parity-structured model of mosquito infection dynamics to consider the comparative effects of different vector control methods. Our results reinforce the important of adult-acting interventions, as well as providing an analytical model framework for quantifying key transmission statistics for vector-borne diseases.

## Introduction

In 2021 there were approximately 247 million cases of malaria worldwide and 619 000 deaths, with children under age 5 accounting for 80% of all fatalities in the World Health Organization (WHO) African Region, which is home to 95% of all malaria cases [1]. Vector control plays a large part of malaria control, with the WHO currently recommending deployment of either long-lasting insecticide treated nets (LLINs) or indoor residual spraying (IRS) in most at-risk areas. Since 2004 over 2 billion nets have been distributed to populations at risk of malaria and their usage has been attributed to 68% of prevented cases in Africa in the 15 years from 2000 to 2015 [2, 3], particularly in pregnant women and children, in whom bednet usage more than doubled (26% to 61%) across this period.

However, over recent years there has been a gradual increase in the incidence rate of malaria, from 230 million cases in 2015 to 247 million in 2021, potentially due to programmatic interruptions during the COVID-19 pandemic [1]. In addition, the use of indoor residual spraying (IRS) has declined, even pre-pandemic, with coverage dropping from 5.8% in 2010 to 2.6% in 2020 [4]. Aside from the effect of the pandemic, this increase in incidence may also be linked to reports from 81 countries describing the development of insecticide resistance [5] or the geographical expansion of viable habits for vector species [1]. Requirements to maintain LLIN efficacy through regular distribution, with each net typically lasting for up to 3 years, or 20 washes, makes control programmes particularly vulnerable to interruptions or discontinuities in provision. Larvicidal methods of control are not currently widely used, but empirical studies have shown using larvicides to reduce mosquito densities can have a positive impact on incidence and malaria parasite prevalence [6]. In particular, potentially due to the similar mechanisms employed by LLINs and IRS, combining adult-acting methods has been shown to have limited additional effect [7], but larval control has been recommended by the WHO as an appropriate supplementary measure to adult vector control.

The existing wide-spread and successful usage of vector control to combat malaria demonstrates the vital role of the mosquito in sustaining transmission. The importance of a deep understanding of how the mechanisms by which these interventions work, as well as the underlying mosquito ecology, has been previously noted [8, 9].

Vector control interventions target the mosquito, impacting the feeding cycle and population characteristics. This means that to understand the mechanism through which vector-based interventions reduce transmission, it is first necessary to understand how vector control measures and coverage affect the vector dynamics. The challenges associated with maintaining vector control interventions also mean that it would be useful to understand how transmission changes with the waning efficacy of these interventions over time. Greater characterisation of the changing dynamics of the mosquito population under vector control would also be vital to any future vector-based surveillance (xeno-monitoring) strategy.

A number of widely used models focusing on transmission dynamics have tended to simplify vector dynamics [10, 11], whereas models that focus on vector dynamics often do not include transmission [9, 12]. Although the historical literature does include good characterisation of the dynamical differences between larval and adult control [13–17], few previous modelling efforts have combined changes in a full range of vector population measures, including abundance and age-structure, with the resulting impact on transmission [18].

Parity, the number of feeding and egg-laying cycles—called gonotrophic cycles—a mosquito has completed, provides a natural discrete measure of age. By constructing a parity-structured mosquito population model of the gonotrophic cycle that also includes mosquito infection status, we aim to further investigate these dynamics. In particular, we consider the effect of different vector control measures on the age- and infection-structure of the vector population and calculate the impact on the reproductive number under control, *R*_*c*_, a key transmission measure that can be used to predict long-term elimination or resurgence [16].

Although vector control methods have long been recognised as important in malaria control and elimination [19], the increasing risk of insecticide resistance and questions around sustainability of interventions have prompted discussions on the best strategy [14, 15, 20, 21]. LLINs are the most commonly used measure, but there is also wide-spread usage of IRS and other insecticidal spraying methods. It has been previously demonstrated that insecticides that target older adult mosquitoes could potentially reduce the sensitivity of vector control interventions to resistance evolution [22]. Although larvicidal methods are often seen as practically more challenging to implement [23], it has also previously been suggested that using multiple interventions at different stages of the life-cycle could give improved results [9, 15, 17], and that it may be easier to target spatially-confined larval stages than their highly mobile adult counterparts [24]. As such, there is need for broader analysis of the relative benefits of different vector control methods and their combinations, including the relationship between mosquito population structure and factors such as intervention efficacy and resistance evolution risk.

Early models of malaria incorporated the mosquito population size, highlighting the importance of the vector to host ratio in determining transmission dynamics [25]. These were extended to derive a formula for the basic reproduction number, *R*_0_, based on this ratio and a number of other variables, including mosquito feeding and survival rates [13]. Recent models have expanded this further to consider the stages of the feeding cycle [26, 27], including vector control interventions at the larval or adult stages but with no age-structure within the adult vector population, with many more models including vector control in some form [28–36]. However, an independent review of 388 mosquito-borne pathogen models found only 5 models that considered any two-intervention combination of LLINs, IRS and larvicides and only one that considered all three [18].

One study used an updated version of Macdonald’s theory of vectorial capacity to consider combinations of larval and adult vector control measures, demonstrating that combined measures could provide an improved ratio of effect size to effort and potentially bring *R*_*c*_ below 1 [15]. However, combining interventions may not always provide additional benefit, with a recent review reporting no detectable changes where pyrethroid-based IRS was implemented in communities using LLINs [7], and relative outcomes could be impacted by the waning efficacy of insecticides over time.

Age-structure has also been modelled explicitly in a number of ways, including splitting the mosquito population into life-cycle stages (egg, larvae, pupae, adult) [37] and focusing on the age-structure of the host population [38, 39]. Partial differential equations (PDEs) have been used to consider adult mosquito age-structure within a transmission model, allowing continuous aging of the mosquito population [40], but this has not been used to compare different vector control measures and their impact on the population structure.

Here we develop and analyse a novel age-structured model of the adult female mosquito population, which allows for consideration of the distribution of how many times a mosquito has taken a bloodmeal, and subsequently the age-dependent infection risk given a particular human prevalence. Inclusion of age-structure, as well as the analytically tractable nature of the model, is a key strength of these methods—providing a model framework that could be generalised to consider other questions, such as the benefit of targeting older mosquitoes to minimise selection pressure, or the relationship between human and mosquito prevalence.

## The model

In this section we described the construction and analytical solution of an age-structured deterministic compartmental model, used to describe the mosquito gonotrophic cycle. The age-structure of the population is measured in the number of gonotrophic cycles, or number of successful bloodmeals, completed by each individual mosquito—otherwise known as the parity of the mosquito. The steady-state of this model is used to investigate the impact of common vector control methods (LLINs, IRS and larvicides) on the age-structure and transmission potential of the vector population.

### Gonotrophic cycle model with vector control

Considering the gonotrophic cycle of an adult mosquito, we divide the stages into four categories: blood-seeking (B), fed (F), gestating (G) and ovipositing (O) [26]. In the absence of intervention new adult mosquitoes are considered to be born into the emerged class at rate *β* and obey a constant natural death rate *g*. Dynamics can then be described using the following system of ordinary differential equations (ODEs):

$$\begin{array}{c} \frac{dB}{dt}=\beta \left(1-\theta \right)+\pi_{1}O-\pi_{2}\left(s+d_{L}\right)B-gB \end{array}$$ (1)

$$\begin{array}{c} \frac{dF}{dt}=\pi_{2}sB-\pi_{3}F-gF \end{array}$$ (2)

$$\begin{array}{c} \frac{dG}{dt}=\pi_{3}\left(1-d_{I}\right)F-\pi_{4}G-gG \end{array}$$ (3)

$$\begin{array}{c} \frac{dO}{dt}=\pi_{4}G-\pi_{1}O-gO\;, \end{array}$$ (4)

where *π*_2_ represents the baseline rate of feeding and moving from blood-seeking to fed; *π*_*i*_, *i* = 1, 3, 4, denote the movement between the other states. The parameters *π*_*i*_ for *i* = 1, …, 4 are chosen to give a 3 day feeding cycle length with 0.68 day mean blood-seeking duration [41]. Lardeux et al. observed a minimum 2 day period for gestation, matching up to an approximate 3 day gonotrophic cycle [42], hence the blood-seeking and gestating stages are assumed to take up the majority of the cycle duration (full details are specified Section A in S1 Text). The magnitude of *β* is used to control the baseline transmission conditions.

Here *s* and *d*_*L*_ represent the probabilities of vector success or death, respectively, during a feeding attempt and *d*_*I*_ is the probability a vector dies after feeding (due to IRS). When no vector control is in use *s* = 1 and *d*_*L*_ = *d*_*I*_ = 0. We consider a successful feed to have occurred in any of three potential scenarios: biting indoors despite LLIN or IRS presence; biting indoors in the absence of LLINs or IRS; and biting outdoors. Biting outdoors is assumed to occur in proportion 1 − *Q*, where *Q* accounts for the human blood index (the proportion of bloodmeals taken on humans) and the probability of biting indoors. Death due to IRS is considered as an additional probability of not surviving between the fed and gestating classes, post feeding and potential transmission. The birth rate is multiplied by a scaling factor (1 − *θ*), where $\theta =\theta_{0}\hat{\theta }$ is a proportional population reduction due to larvicides; *θ*_0_ is the coverage (i.e. proportion of larval sites treated) and $\hat{\theta }$ is the efficacy of the intervention, or proportional reduction in adult mosquitoes emerging from a treated larval site.

**Fig 1:**
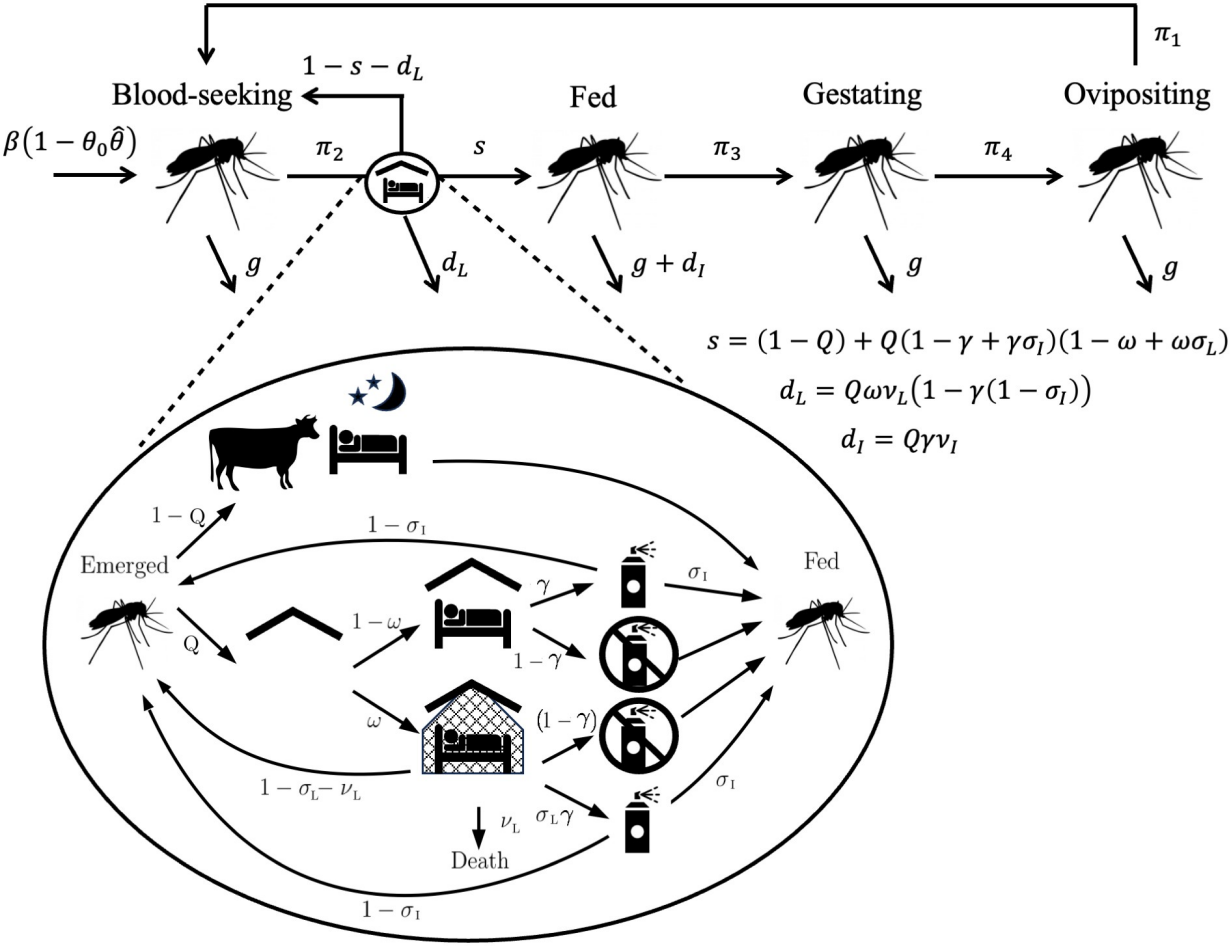
Gonotrophic cycle with vector control. A schematic depicting the mosquito gonotrophic cycle model. In the model mosquitoes move from Blood-seeking (B), to Fed (F), to Gestating (G), to Ovipositing (O) and back to Blood-seeking. Larvicide usage impacts the emergence, or birth, rate of adult mosquitoes and LLIN and IRS interactions take place between Blood-seeking and Fed. All icons used have been made available under the Creative Commons CC0 1.0 Universal Public Domain Dedication via Wikimedia Commons.

The values of *q*_*i*_, *i* = 1, …, 3 are given by the following equations, calculated using the feeding dynamics described by Fig 1,

$$\begin{array}{c} s=\left(1-Q\right)+Q(1-\gamma +\gamma \sigma_{I})(1-\omega +\omega \sigma_{L})\;, \end{array}$$ (5)

$$\begin{array}{c} d_{L}=Q\omega \nu_{L}(1-\gamma (1-\sigma_{I}))\;, \end{array}$$ (6)

$$\begin{array}{c} d_{I}=Q\gamma \nu_{I}\;, \end{array}$$ (7)

with *ω* and *γ* representing the coverage of LLINs and IRS respectively. *σ*_*L*_ and *ν*_*L*_ are the success and death probabilities of feeding in the presence of an LLIN, where 1 − *σ*_*L*_ − *ν*_*L*_ is the probability of repelling. *σ*_*I*_ is the probability of successfully feeding in the presence of IRS, where 1 − *σ*_*I*_ is the probability of repelling, and *ν*_*I*_ is the probability of death during the Fed class immediately after exposure to IRS. Values of all parameters are given in Tables B-D and Section A, in S1 Text.

### Age structure

To gain insight into the age-structure of the vector population we consider a generational formulation of the gonotrophic cycle model, where a subscript *i* denotes the number of times mosquitoes in a given class have completed the cycle, initially giving an infinite system of ODEs:

$$\begin{array}{ccc} \frac{dB_{i}}{dt} & = & \left\{ \begin{array}{cc} \beta \left(1-\theta \right)-\pi_{2}\left(s+d_{L}\right)B_{i}-gB_{i} & \;\text{if}\;i=0\\ \pi_{1}O_{i-1}-\pi_{2}\left(s+d_{L}\right)B_{i}-gB_{i} & \;\text{if}\;i\geq 1 \end{array}\right. \end{array}$$ (8)

$$\begin{array}{c} \frac{dF_{i}}{dt}=\pi_{2}sB_{i}-\pi_{3}F_{i}-gF_{i} \end{array}$$ (9)

$$\begin{array}{c} \frac{dG_{i}}{dt}=\pi_{3}\left(1-d_{I}\right)F_{i}-\pi_{4}G_{i}-gG_{i} \end{array}$$ (10)

$$\begin{array}{c} \frac{dO_{i}}{dt}=\pi_{4}G_{i}-\pi_{1}O_{i}-gO_{i}\;. \end{array}$$ (11)

Births can only occur into generation *i* = 0 and, to avoid inclusion unrealistically old vectors, we make the simplifying assumption that vectors can only survive up to a maximum of 10 gonotrophic cycles [43]. This reduces our infinite system of ODEs to a finite system, allowing values of *i* = 0, 1, …, 10. Using this model it is possible to calculate the parity of the population.

A frequently used assumption in modelling vector borne diseases is that the vector population can be considered to be at equilibrium relative to the human population at any point in time [44, 45]. This is due to the relative mosquito lifespan being approximately 500 times shorter than that of a human, creating a multi-scale problem that allows the application of a quasi-steady state assumption on the mosquito dynamics—meaning we assume mosquito population size, structure, and infection profiles will respond instantly to any change in the system and adjust to a new equilibrium. Here we don’t include an explicit model of human dynamics, but the quasi-steady state solution of this model could be built into a full transmission model.

The birth rate of mosquitoes in this model only affects the overall magnitude of the population distribution, therefore the quasi-steady state assumption, combined with our key interest lying in the proportion of mosquitoes in each generation and the prevalence of infection, allows us to assume a constant birth rate, *β*. We can later consider the effects of population magnitude through the use of the vector host ratio—a commonly used parameter that describes the relative size of the vector and human populations.

We can hence derive the following relationship between sequential blood-seeking classes by setting Eqs 8–11 to zero and rearranging:

$$\begin{array}{c} B_{i}^{*}=KB_{i-1}^{*}\;, \end{array}$$ (12)

where

$$\begin{array}{c} K=\frac{\pi_{1}\pi_{2}\pi_{3}\pi_{4}s\left(1-d_{I}\right)}{\left(\pi_{2}\left(s+d_{L}\right)+g\right)\left(\pi_{3}+g\right)\left(\pi_{4}+g\right)\left(\pi_{1}+g\right)} \end{array}$$ (13)

is a constant and *K* < 1 as at equilibrium each generation will be smaller than the previous younger generation, with the newly emerged generation being the largest. This quantity, *K*, can also be interpreted as the gonotrophic cycle survival probability, or the proportion of the vectors that are gravid (have had at least one bloodmeal). As the constant term is less than unity, the difference equation can be solved to get an explicit formula,

$$\begin{array}{c} B_{i}^{*}=K^{i}B_{0}^{*}\;, \end{array}$$ (14)

which can be used to calculate the number of vectors in each feeding generation for initial conditions

$$\begin{array}{c} B_{0}=\frac{\beta (1-\theta )}{\pi_{2}\left(s+d_{L}\right)+g}\;. \end{array}$$ (15)

The proportion of the population that have completed at least one feeding cycle (are parous) is given by

$$\begin{array}{c} 1-\frac{B_{0}}{\sum_{i}B_{i}}\;. \end{array}$$ (16)

### Vector infection model

**Fig 2:**
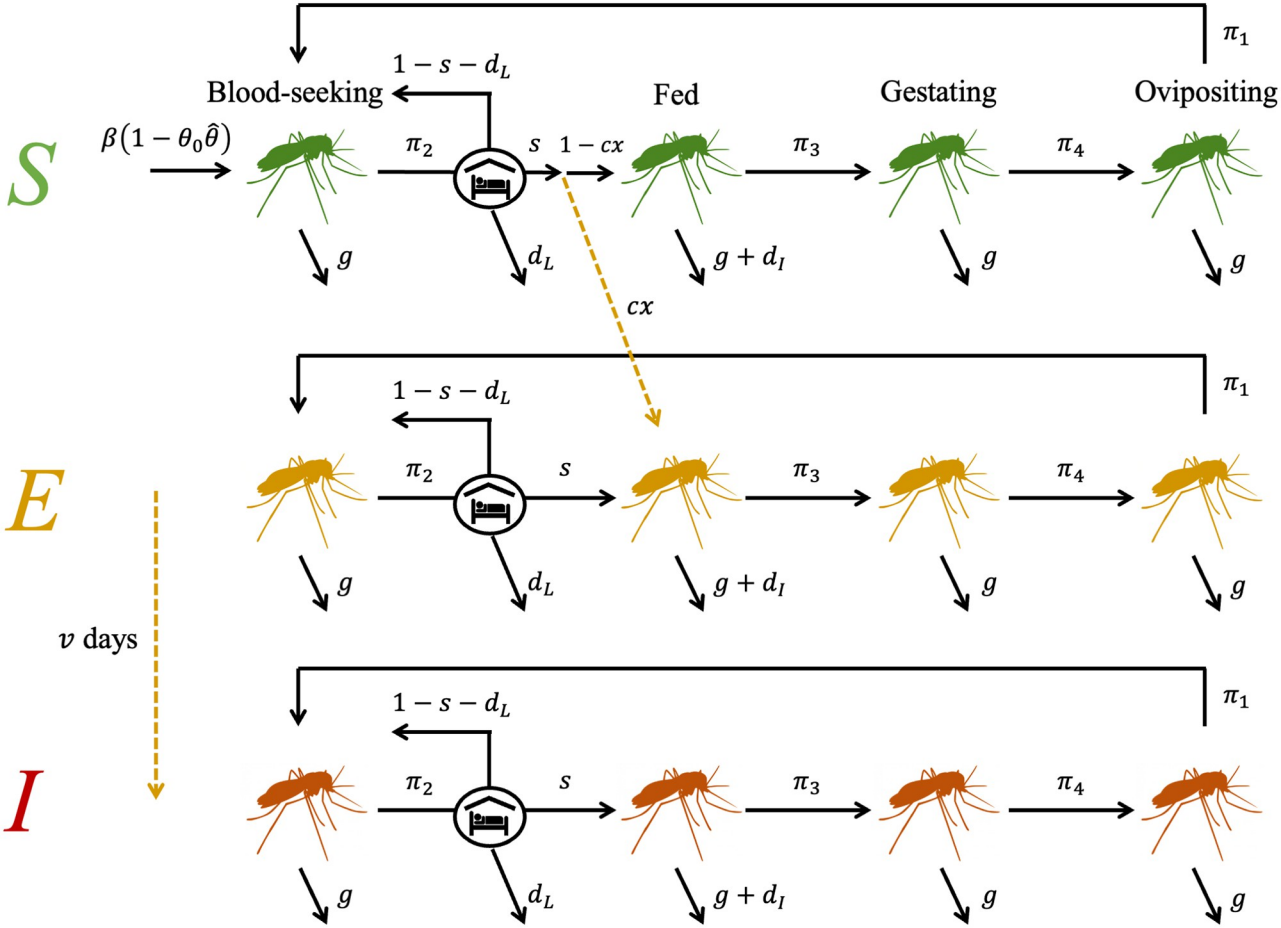
SEI disease model in mosquitoes. A schematic depicting the SEI (susceptible, exposed, infectious) formulation of the disease model used. Upon successful feeding, mosquitoes become infected with probability *p* and enter the exposed class. Mosquitoes transition from exposed to infectious at rate 1/*ν*, where *ν* is the extrinsic incubation period of malaria. All icons used have been made available under the Creative Commons CC0 1.0 Universal Public Domain Dedication via Wikimedia Commons.

We consider a standard SEI model for the vector population with three disease states: susceptible (*S*), exposed (*Y*) and infectious (*Z*). Extending the ODE model (as in Eqs 1–4) to include disease requires sub-dividing each stage of the cycle into these three states, giving a new system of twelve ODEs for each generation, *i*. We assume births only occur in the susceptible population and in generation *i* = 0V. Assuming a prevalence *x* in the human population and a probability *c* that a vector becomes infected after biting an infectious human, then a proportion *xc* of susceptible vectors moving from blood-seeking to fed become exposed to disease. Considering a mean extrinsic incubation period (EIP) in the vector of *v* days, for analytical tractability we choose to discretise this period to the number of cycles this would take when evaluating our model. For example, for a cycle length of 3 days and a mean EIP of 10 days, this would mean a vector is expected to become infectious during the 4th cycle post-exposure, allowing for a range of between 9 and 12 days. Due to timescales of infection and vector lifespan we do not consider recovery from infection. See Fig 2 for a diagram of the full model dynamics and Table E and Section B, in S1 Text for the disease parameter values used.

As the host dynamics are slow in comparison to the vector dynamics, we assume that for any change in host prevalence the vector population reaches equilibrium in negligible time. Hence we can use the equilibrium state of the model as an approximation for the age and disease distributions for any given human prevalence and use these to calculate transmission measures commonly used in vector-borne disease epidemiology, such as the entomological inoculation rate (EIR), which can be estimated using field data [46, 47].

### Entomological inoculation rate (EIR)

The entomological inoculation rate (EIR), *E*, is the expected number of infectious bites received by a single host across a defined time period, described by

$$\begin{array}{c} E=maz\;, \end{array}$$ (17)

where *m* is the ratio of mosquitoes to humans, *a* is the blood-feeding rate on humans, both of which depend on vector control measures, and *z* is the fractional prevalence of infectious vectors given by:

$$\begin{array}{c} z=\frac{K^{N}B_{0}\sum_{i=0}^{n}\left[K^{i}-K^{i}{\left(1-\kappa \right)}^{i}\right]}{\sum_{i=0}^{n}B_{i}} \end{array}$$ (18)

The full derivation of *z* and expressions for *a* and *m* are provided in Section B in S1 Text.

### Vectorial capacity

Vectorial capacity, *V*, denotes the total number of infectious bites that would eventually arise from all the mosquitoes that bite a single infectious human on a single day [48].

$$\begin{array}{c} V=\frac{ma^{2}p^{v}}{-\text{ln}(p)}=\frac{ma^{2}}{g}e^{-gv}\;, \end{array}$$ (19)

where *p* is the vector daily survival probability and *v* is the extrinsic incubation period in the vector. Alternatively, *g* is the instantaneous vector death rate. Both *p* and *g* will depend on vector control measures, with full expressions given in the Section B in S1 Text.

### Basic reproductive number

From the vectorial capacity we can derive the basic reproductive number under control, *R*_*c*_, for vector borne diseases. This differs from the usual interpretation of *R*_*c*_ for non-vector diseases by focusing on the vector dynamics, describing the number of new infectious mosquitoes that would arise from a single infectious mosquito after one parasite generation [48].

$$\begin{array}{c} R_{c}=\frac{ma^{2}bc}{gr}e^{-gv}=\frac{ma^{2}bc}{-\text{ln}(p)r}p^{v}=\frac{Vbc}{r}\;, \end{array}$$ (20)

where *b* is the probability a bite from an infectious vector infects a human, *c* is the probability a bite on an infectious human infects a vector, and *r* is the human disease recovery rate. Under the assumption that these three parameters are approximately constant, *R*_*c*_ is hence linearly proportional to vectorial capacity.

### Vector control

Vector control interventions impact the vector population size, and hence the mosquito to human ratio, they also affect vector prevalence, feeding cycle length and death rate. Using our model to characterise these relationships for a range of coverages, we can directly calculate the aforementioned transmission measures in the presence of LLINs, IRS or larvicides, as well as any combination of the three (see Section B in S1 Text for analytical derivations and functional forms).

Data describing the efficacy of different adult-acting vector control interventions were taken from a range of sources, including a systematic review of IRS efficacy in Africa [49, 50]. In this study we also vary the condition of LLINs, both in structural integrity [49] and insecticide waning effects (assuming a 2 year half-life [51]). We will consider IRS with pyrethroids in the primary instance, but also present some results for organophosphates as a comparison [50]. Larvicidal usage is conservatively assumed to provide up to a 60% reduction in the adult mosquito population, [52], dependent on coverage, although the true magnitude of this effect will likely be highly setting- and species-dependent.

## Results

The model derivation and analyses outlined in the Methods are the major focus of this paper, in particular the explicit quasi-equilibrium solution described by Eqs (12) to (16). Here, we briefly present the impact of interventions and changes in vector dynamics on the age-structure of the vector population and the transmission of disease using these expressions. The model is presented here in the context of malaria but could easily be extended to consider other mosquito-borne diseases by adjusting the disease-specific parameters—although if vertical transmission (from mosquito to offspring) is possible, then consideration of this may need to be included in the infection model.

**Fig 3:**
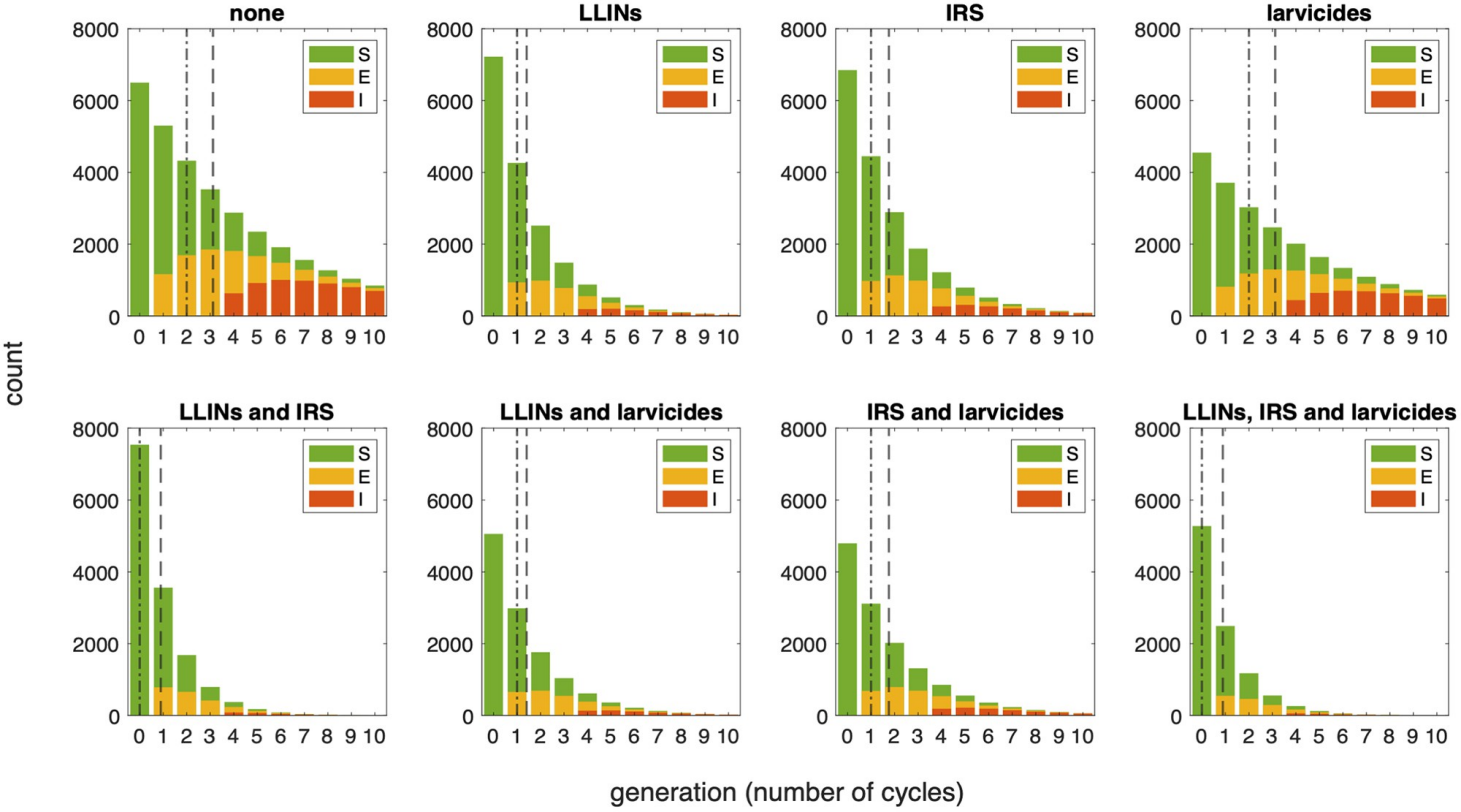
Population age-distribution with disease. Bar plots showing the age-distribution of a vector population at equilibrium (total count, indexed by number of gonotrophic cycles completed) with a variety of vector control interventions (top row) and combinations (bottom). All interventions are assumed to have 50% coverage. Bars are coloured by the proportion of vectors in each cycle generation that are susceptible (green), exposed (yellow) and infectious (red) for malaria at 40% host prevalence. Vertical lines represent the mean (dashed) and median (dot-dashed) number of gonotrophic cycles a mosquito passes through before dying.

As has been noted previously, both LLIN and IRS usage have a two-pronged effect on the vector population. Repelling vectors from feeding extends the blood-seeking phase, decreasing the frequency of bloodmeals per vector. In addition, the insecticidal effects also reduce the total population size by killing vectors either before (LLIN) or after (IRS) feeding; the main killing effect of IRS occurs after feeding as blood-fed mosquitoes typically find somewhere to rest immediately post-feed, often on internal walls that may have been treated with IRS. These effects both act to decrease the number of average bloodmeals per vector per lifetime, hence reducing the likelihood of successful contraction, incubation, and transmission of disease. Considering the vector population structure, this manifests as a smaller total population with the age-distribution shifted towards the younger generations (Fig 3).

Conversely, larvicides work by targeting larval stages and hence reducing the adult emergence rate. This results in a reduction in overall population size but doesn’t impact the behaviour or habits of the vectors once they have developed to adulthood, or their individual transmission potential. Our model therefore predicts a linear impact on population size and vector prevalence and doesn’t impact the shape of the generational distribution (also Fig 3).

**Fig 4:**
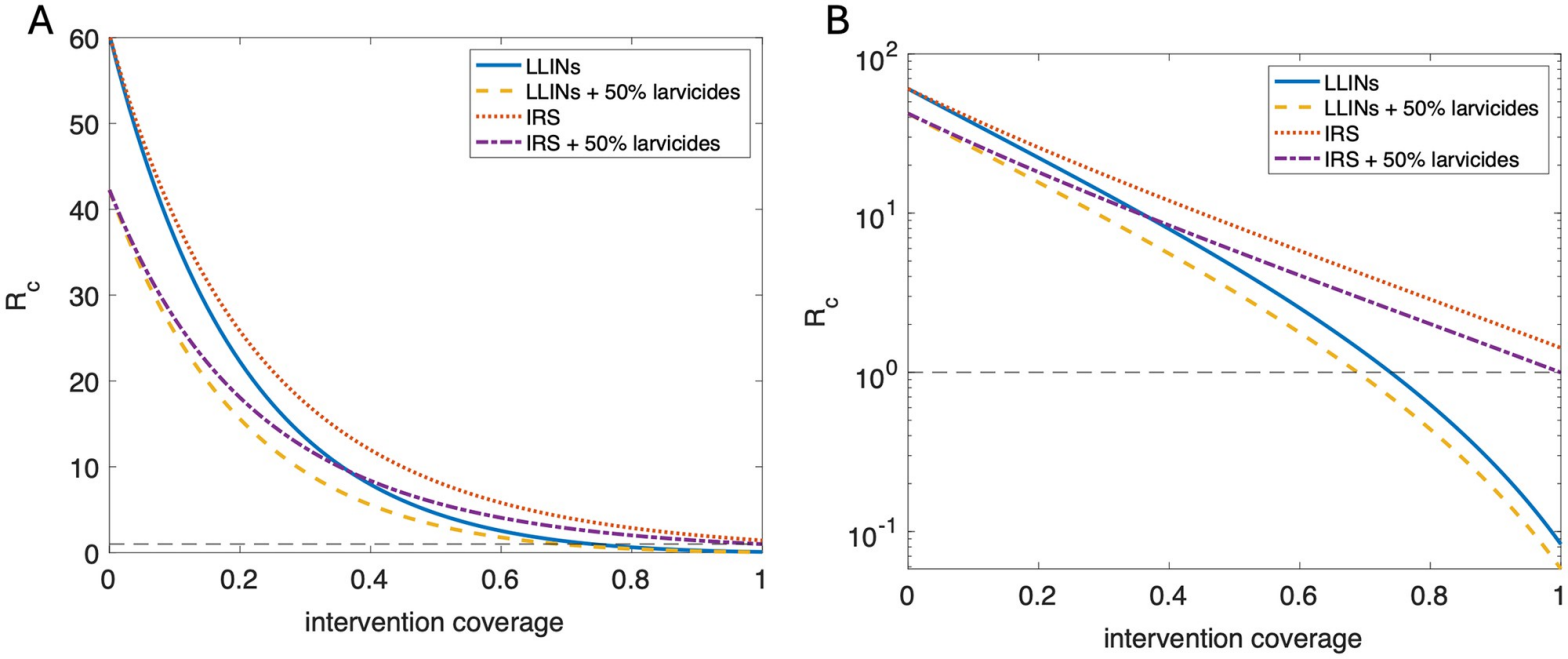
Reproductive ratio, *R*_*c*_, by vector control method and coverage. Graphs showing the relationship between *R*_*c*_ and coverage for the two adult-acting vector control interventions, with or without additional 50% coverage of larvicides: LLINs (solid, blue); LLINs and larvicides (dashed, yellow); IRS (dotted, red); IRS and larvicides (dot-dashed, purple). Left: linear y-axis; Right: logarithmic y-axis. All results for a mid-to-high transmission setting (*R*_0_ = 60).

LLINs mostly act by repelling or killing vectors pre-feeding, meaning the effect on *R*_*c*_ scales up faster with coverage than that of IRS, which mostly kills vectors post-feeding (Fig 4). The addition of larvicides to either of these measures reduces the coverage required to bring *R*_*c*_ below 1 (see Table 1). In particular, even an unrealistic assumption of 100% coverage of pyrethroid-based IRS (representing every wall in every house being treated) doesn’t bring *R*_*c*_ below 1 unless implemented in combination with high coverage larvicidal usage.

**Table 1:** Vector control coverage combinations required to bring *R*_*c*_ < 1.

Larvicides	LLINs (6 holes)	LLINs (80 holes)	IRS (pyrethroids)	IRS (organophosphates)
None	74%	96%	NA	56%
50%	69%	91%	100%	51%
100%	61%	80%	84%	43%

For a setting where *R*_0_ = 60. Larvicidal percentages reflect proportion of larval sites treated.

**Fig 5:**
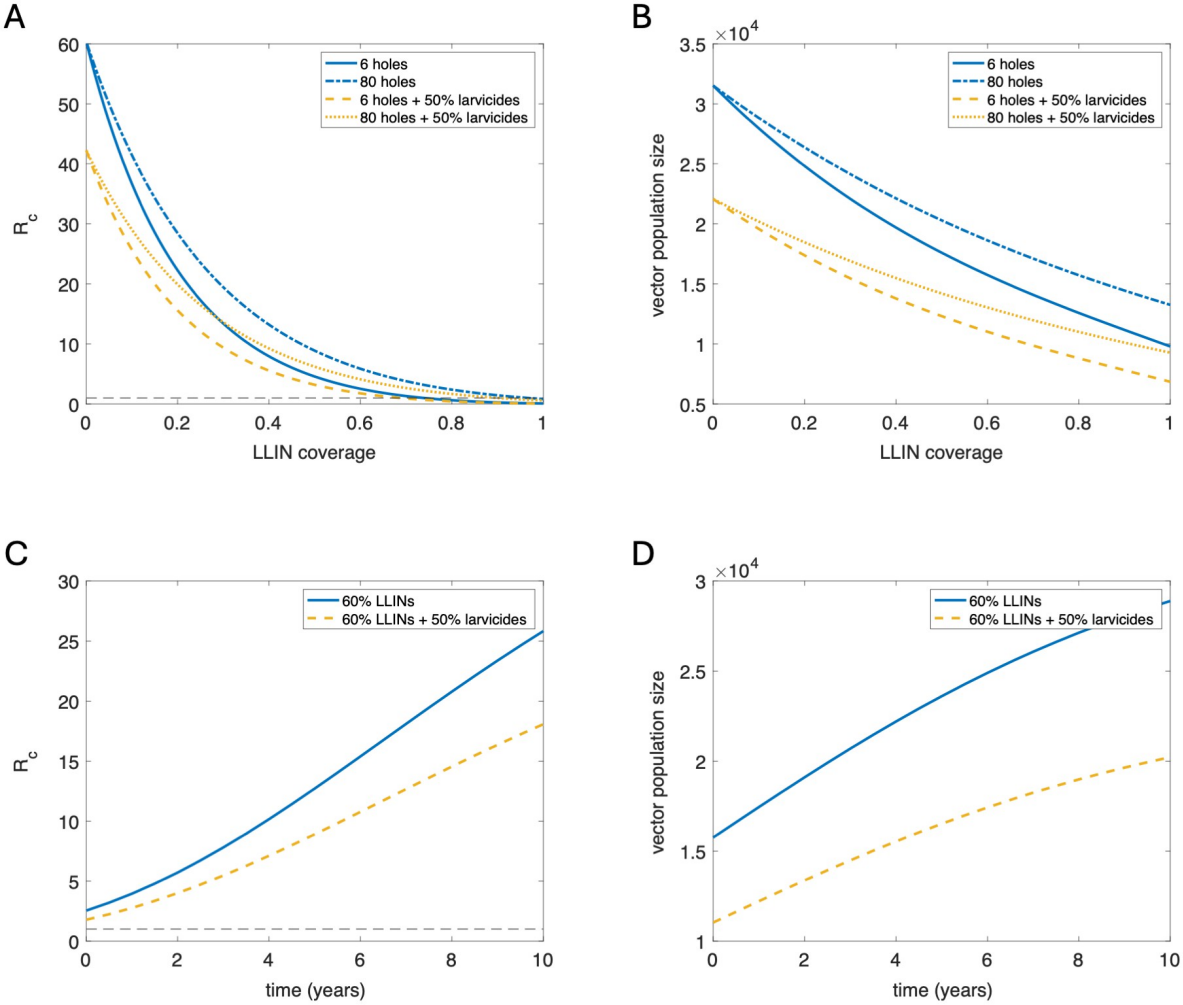
LLIN integrity and waning insecticidal effects. Top row: Graphs showing the relationship between *R*_*c*_ (left) and vector population size (right) and LLIN coverage for good condition nets (6 holes: solid, blue; 6 holes plus 50% larvicides: dashed, yellow) and poor condition nets (80 holes: dot-dashed, blue; 80 holes plus 50% larvicides: dotted, yellow). Bottom row: Graphs showing changes in *R*_*c*_ and vector population size over time due to waning insecticidal efficacy with an assumed half life of 2 years, for LLINs only (solid, blue) and LLINs with 50% larvicides (dashed, yellow). All results for a mid-to-high transmission setting (*R*_0_ = 60).

Mid-ranges of LLIN coverage see the largest effect of reduced integrity on the effective reproductive ratio, *R*_*c*_, a key measure of transmission (Fig 5: top). However, the difference between vector population size grows wider as coverage increases. Using larvicides at 50% coverage in combination with LLINs can mitigate most of these effects, based on our assumptions around larvicide efficacy, with combined usage of poor condition (80 hole) LLINs and larvicides performing better than good condition (6 hole) LLINs used in isolation for low coverages of up to almost 30%.

Using larvicides with LLINs can also help to slow the effect of waning insecticidal efficacy on transmission over time by helping to keep the vector population size down (Fig 5: bottom). However, even at high coverages these reductions in efficacy may still undermine program outcomes if new LLINs are not distributed sufficiently frequently.

## Discussion

The model developed here provides an easy-to-use analytical framework for investigating the complex interactions between vector control interventions, age-structured vector population dynamics and disease transmission. It could be extended in a number of ways, including further analysis of the impact of waning interventions, as demonstrated in Fig 5, or the increase of insecticidal resistance.

Our results demonstrate that LLINs and IRS are more effective than larvicidal interventions, and that the gap in utility increases with increasing coverage, but that larval control can still play an important role in maximising programme impact. The effect of LLINs and IRS is closely comparable at low coverage, but as coverage increases LLINs have an progressively larger impact than IRS on transmission. Additionally, in medium to high transmission settings the slower decrease in *R*_*c*_ with increasing IRS coverage is insufficient to break transmission, even for an unrealistic 100% coverage, if not acting in combination with other measures. Although our results suggest high levels of LLIN usage (≥74%) could bring *R*_*c*_ below one in the scenario considered, heterogeneous biting patterns may undermine this effect [53]. Larvicides may therefore be able to play a role in sustaining, or even advancing, existing gains [54, 55], particularly in low transmission settings where incidence has been brought down artificially through other interventions.

The definitions of coverage used (percentage of individuals sleeping under LLINs, houses sprayed, or larval breeding sites treated) are difficult to compare across interventions, particularly in terms of the associated costs and feasibility. It has been suggested that LLINs may require less effort to scale up coverage in the 40–80% coverage region [15], but coverage measures often exclude considerations of adherence. Additionally, we have only considered night biting mosquitoes in our analysis and in a setting with high proportions of day biting mosquitoes we would expect LLINs and IRS to have a reduced effect, whilst larvicidal impact should be mostly unchanged.

For larvicidal coverage it would be impossible to find and treat every breeding site if the area considered wasn’t very small, and would require regular maintenance [52], meaning that achieving high coverage in the terms described here is likely to be difficult in practice, although there has been some progress in developing slow-release larvicides, with the potential for effects lasting closer to 6 months to a year [56]. Even if regular maintenance is required, larvicidal use can be instilled locally without reliance on large funders and the impact doesn’t depend on population adherence or mosquito feeding behaviours, making it an attractive option for programmes looking for supplementary control measures. However, we have only considered a limited range of efficacy for larvicides, based on a single study from a specific setting, and more investigation is required before any concrete recommendations around larvicidal usage can be made.

We have made a number of additional assumptions about the mosquito biology, including that infection has no impact on vector fitness and that mortality remains constant with age (up to a maximum life span). This second assumption is consistent with current understanding of wild mosquito populations; although senescence is observed in laboratory mosquitoes, wild mosquitoes are expected to die long before they can exhibit any substantial deterioration with age [57]. Our current understanding of the first assumption is broadly inconclusive, with conflicting views and results across the literature [58]. These assumptions, and the resulting estimated mosquito age-distribution, can be logistically challenging to validate, currently relying on complex dissection methods to determine parity and the number of gonotrophic cycles a mosquito has completed [59]. However, there is hope that emerging technologies, such as transcriptional profiling and mid-infrared spectroscopy, may simplify this process in the future [60].

Our model doesn’t presently take account of seasonal changes in vector population sizes and behaviours, which is known to have a substantial effect on malaria transmission [61]. At lower temperatures both the extrinsic incubation period and gonotrophic cycle are expected to take longer, with our parameters reflecting temperatures of approximately 28°C or higher [42, 62]. For regions where temperatures drop below this level for a sustained period of time the extended incubation and cycle lengths should lead to reduced transmission, meaning our results are more reflective of high season transmission.

It is also important to remember that scale-ups in use of insecticides to combat transmission can result in wide-spread insecticide resistance and behavioral changes in sleeping conditions can lead to changes in biting behavior [21, 63, 64]. These factors have the potential to undermine progress made using vector control measures, and in particular evidence of this has been seen in a number of malaria control programs [65–67]. This is less of a problem for larvicidal interventions, which are less widely used and have a wider range of chemical and biological agents [68]. Settings where resistance has been observed or is feared may benefit from a combination of interventions, in particular larvicides could be used to accelerate gains and delay resistance by slowing the vector birth rate [55].

This age-structured model has multiple potential extensions and applications beyond considering the effect of vector control on mosquito population structure and key epidemiological metrics. Explicitly modelling the vector population structure could potentially improve the utility and accuracy of existing vector-borne disease transmission models [69], as well as supporting characterisation of the relationship between vector and human prevalence, which is crucial for the interpretation of xeno-monitoring (vector-based surveillance) data. Linking these methods with previous work on the evolution of resistance to vector control interventions [22] could also help to inform the targeting of insecticidal methods to minimise resistance risk whilst maximising transmission reduction.

## Conclusions

Whilst well-maintained adult-acting vector control measures are substantially more effective than larval-based interventions if used in isolation, incorporating larval control in existing LLIN or IRS programmes could substantially reduce transmission. This would most benefit areas with low coverage or poor maintenance of interventions, or where insecticide resistance means that LLINs and IRS have reduced efficacy.

Additionally, the model framework developed here is adaptable to different species of mosquito and would be easily extended to consider a number of other mosquito-borne infections through a change of parameterisation. The utility of explicitly describing the impact of different interventions on the vector population size and structure could therefore assist with developing a greater understanding of vector ecology and epidemiology, which could be beneficial in developing future control and surveillance methods and planning for the impact of insecticide resistance.

## Supporting information

S1 Text Further methods. Full analytical model derivations, functional forms of vector-control dependent transmission measures, and parameter estimates and sources.
(PDF)
