# Supplementary material for: An analytically tractable, age-structured model of the impact of vector control on mosquito-transmitted infections
Source: PLoS Comput Biol. 2024 Mar 14;20(3):e1011440. doi: 10.1371/journal.pcbi.1011440 (PMC10965057; doi:10.1371/journal.pcbi.1011440)
Supplement: S1 Text — Full analytical model derivations, functional forms of vector-control dependent transmission measures, and parameter estimates and sources. (PDF) [file pcbi.1011440.s001.pdf]

S1 Text: An analytically tractable, age-structured model of the impact of vector control on mosquito-transmitted infections.

Emma L Davis[1,2*], T Déirdre Hollingsworth[3] and Matt J Keeling[2]

[1*]Mathematics Institute, University of Warwick, Coventry, UK.
[2]Zeeman Institute, University of Warwick, Coventry, UK.
[3]Big Data Institute, University of Oxford, Oxford, UK.

*Corresponding author(s). E-mail(s): Emma.L.Davis@warwick.ac.uk;

Section A: Parameterisation

Vector parameters

In order to consider interventions in terms of coverage, we define the action of these three measures as described in Table A. We consider coverage of LLINs to describe the percentage of indoor-sleeping individuals who sleep daily under bednets; coverage of IRS describes the percentage of people who have had their bedrooms sprayed in the previous 6 months; coverage of larvicides is taken to be the percentage coverage (by area) of larval sites with weekly larvicidal treatment.

An experimental hut trial in Benin tested the efficacy of LLIN and IRS interventions using a pyrethroid-impregnated polyester LLIN, untreated nets and chlorfenapyr IRS [1] against *Anopheles gambiae* and *Culex quinquefasciatus*. The LLINs used were deliberately provided with either 6 holes (4cm^2 each) or 80 holes (2cm^2 each) to simulate different levels of integrity. The results for *Anopheles gambiae* vectors are shown in Tables B and C. LLINs were found to have the highest repelling effect, with only 12.1% of vectors successfully feeding in the presence of a bednet. In comparison, untreated nets allowed 56.4% of vectors to successfully feed (see Table B). The insecticide used was assumed to have a half-life of 2 years [2], allowing variation of repelling and mortality parameters from those observed for new LLINs (6 holes, insecticide-treated) to those observed for untreated nets.

Table A Vector control coverage definitions

Control measure	50% coverage	100% coverage
LLINs	50% hosts sleep under nets	All hosts sleep under nets
IRS	50% bedrooms sprayed	All bedrooms sprayed
larvicides	50% larval sites treated	All larval sites treated

Table B Adult vector control: outcome probabilities from feeding attempts in the presence of LLINs [1].

Control measure	Feed success	Death (pre-feed)
LLINs (6 holes)	0.121 (0.054–0.188)	0.495 (0.392–0.597)
LLINs (80 holes)	0.318 (0.231–0.405)	0.373 (0.282–0.463)
Untreated nets	0.564 (0.454–0.674)	0.051 (0.002–0.100)

Table C Adult vector control: outcome probabilities from feeding attempts in the presence of IRS, taken from a systematic review of IRS efficacy in Africa [3].

IRS type	Death post-feed	Deterrence	Feeding inhibition
Pyrethroids	0.44 (0.05–0.93)	0.06 (-1.00–0.77)	0.18 (-0.30–0.83)
Organophosphates	0.88 (0.40–1.00)	0.42 (-1.00–0.95)	0.07 (-0.18–0.49)

IRS parameters for deterrence (pre-entering), feeding inhibition and death (post-feed) were taken from a review study looking at the efficacy of IRS in Africa (see Table C).

Use of the biological larvicide *Bacillus thuringiensis israelensis* (Bti) to treat *Anopheles* breeding sites has been tested in a study in Peru and Ecuador [4]. The larvicide was found to be effective, but due to the surface feeding habits of *Anopheles* larvae, it was found to be only effective for the first 7-10 days following spraying, after which it had sank sufficiently below the surface to have no further impact. The study saw an average adult density reduction (measured in bites per person per hour) of 50 - 70% in the 7 days following treatment across all identified larval breeding sites in a 2km radius. In the model we assume sustained larvicidal coverage provides up to a 60% reduction in the adult population – included as a reduction in the emergence rate and scaled by the coverage, or proportion of larval sites treated, where 100% coverage results in a 60% reduction. This is likely to be a conservative estimate, as this assumes the reported reductions of 50-70% are linked to perfect coverage, but the magnitude of larvicidal impact will be setting- and species-dependent.

Table D contains a summary of the vector-specific parameters, including feeding and biological parameters as well as vector control intervention assumptions. The

Table D Parameters for mosquito biology and vector control (*Anopheles gambiae*).

	Definition	Value	Source
Q	Fraction of blood-meals on humans	0.9-0.95	[5]
π_2	Daily rate of feeding when blood-seeking	1/0.68	[5]
$1/\pi_1$	Ovipositing period	0.16 days	[6]
$1/\pi_3$	Feeding period	0.16 days	[6, 7]
$1/\pi_4$	Gestation period	2 days	[6]
δ	Mean feeding cycle length	3 days	[5]
a	Daily rate of feeding on humans	Q/δ	[8]
g	Natural daily death rate	1/14	[9, 10]
n	Maximum number of cycles a vector can live for	10	[11]
$\hat{\theta}$	Proportion of larvae that die from larvicidal treatment	0.6 (0.5-0.7)	[4]
β	Adult mosquito emergence rate from larval stages	1000-100000 (dependent on disease and setting)	

parameters π_i for $i = 1, ..., 4$ are chosen to give a 3 day feeding cycle length with 0.68 day mean blood-seeking duration [5]. Lardeux et al. observed a minimum 2 day period for gestation, matching up to an approximate 3 day gonotrophic cycle [7], hence the blood-seeking and gestating stages are assumed to take up the majority of the cycle duration. Feeding and ovipositing periods are therefore short in comparison and are set to be equal to make up the remainder of the cycle period in the absence of further evidence ($1/\pi_1 = 1/\pi_3 = 0.16$).

Disease parameters

Table E shows the values used for disease-specific parameters, such as the intrinsic and extrinsic incubation periods and the mean human infection duration.

Table E Disease parameters for malaria (*Plasmodium falciparum*).

	Definition	Value	Source
c	Proportion bites on infectious humans that result in mosquito infection	0.55 (0.47-0.63)	[12]
b	Proportion bites from infectious mosquitoes that result in human infection	0.037 (0.018-0.055)	[5]
u	Intrinsic incubation period in humans (days)	12 (8-23)	[13]
v	Extrinsic incubation period in vector (days)	10 (10-21)	[14]
$1/r$	Mean human infection duration (days)	14	[9]
x	Prevalence of infection in human population	varied	
κ	Probability vector infected after blood meal	cx	

Section B: Derivations of vector control dependent transmission measures

Consider the age-structured gonotrophic cycle model at equilibrium, such that the number of blood-seeking vectors in generation i is $K^i B_0$, where

$$K = \frac{\pi_1\pi_2\pi_3\pi_4 s}{(\pi_2(s+d_L)+g)(\pi_3+g+d_I)(\pi_4+g)(\pi_1+g)} \tag{A}$$

and

$$B_0 = \frac{\beta(1-\theta)}{\pi_2(s+d_L)+g}\,. \tag{B}$$

It is sufficient to consider the blood-seeking class as this is the stage of the feeding cycle where vectors have potential to pick up or transmit disease through biting. If we define the binomial probability, $\kappa = xc$, of a successful feed leading to a new vector infection, then the probability a mosquito becomes exposed during generation i is $\kappa^{(i)} = (1-\kappa)^{i-1}\kappa$. Hence the probability a mosquito has been exposed before generation i is $[1-(1-\kappa)^i]$. Combining these gives the number of vectors in generation n that are already infected:

$$B_0 K^i [1-(1-\kappa)^i]\,. \tag{C}$$

The total number of diseased, D (exposed, Y, or infectious, Z), vectors is given by summation across the generations;

$$D = B_0 \sum_{i=0}^{n} [K^i - K^i(1-\kappa)^i] \tag{D}$$

$$\approx B_0 \frac{K[(K-1)(1-\kappa)^{n+1}K^n + (1-K+\kappa K)K^n - \kappa]}{(K-1)(1-K+\kappa K)}\,, \tag{E}$$

assuming n is the maximum number of generations a vector may live for.

If the incubation period is assumed to be equivalent to N generations (or cycles), then the probability of surviving until infectious is given by K^N and can be treated as a multiplicative factor when calculating the numbers of infectious and exposed vectors:

$$Z = K^N D\,, \tag{F}$$

$$Y = D - Z\,. \tag{G}$$

and hence directly calculate transmission measures as discussed in the main text.

In the absence of interventions the mean feeding cycle length is,

$$\delta = 1/\pi_1 + 1/\pi_2 + 1/\pi_3 + 1/\pi_4\,, \tag{H}$$

where $1/\pi_2$ is the average time to hunt and take a blood meal. IRS and larvicides won't impact hunting time, but the repelling effect of bednets will result in some vectors taking longer to move from emerged to fed.

If we assume a repelled vector begins the hunting process from scratch, then the expected time taken to successfully feed will be equal to the time taken to feed given a successful first attempt plus the expected time taken to feed scaled by the proportion of vector that repeat on any given attempt.

$$\mathbb{E}[\text{Time to feed}] = \mathbb{E}[\text{Time} \mid \text{Successful attempt}] \quad \text{(I)}$$
$$+\mathbb{P}[\text{Repeat}]\mathbb{E}[\text{Time to feed}] \quad \text{(J)}$$
$$\mathbb{E}[\text{Time to feed}] = \frac{1}{\pi_2} + Q\omega(1-\sigma-\nu)\mathbb{E}[\text{T to feed}] \quad \text{(K)}$$
$$\mathbb{E}[\text{Time to feed}] = \frac{1}{\pi_2(1-Q\omega(1-\sigma-\nu))} \quad \text{(L)}$$
$$\text{(M)}$$

Now we can express overall feeding cycle length, δ, in terms of bednet parameters:

$$\delta = \frac{1}{\pi_2(1-Q\omega(1-\sigma-\nu))} + \frac{1}{\pi_3} + \frac{1}{\pi_4} + \frac{1}{\pi_1} \quad \text{(N)}$$

and the human blood feeding rate is given by:

$$a = \frac{Q}{\delta} \quad \text{(O)}$$

The ratio of vectors to humans m, can be scaled by changes in the mosquito population ($m = M/H$), where

$$M = \sum_{i=0}^{n} B_0 K^i = \frac{B_0(1-K^{n+1})}{1-K} \quad \text{(P)}$$

and K describes the probability of surviving each feeding cycle, with dependence on vector control parameters included in B_0 and K.

The death rate will depend on IRS and bednet usage. We can relate the probability of a vector surviving one feeding cycle, K, to a per cycle death rate $-ln(K)$, then we have

$$g = \frac{-ln(K)}{\delta} \quad \text{(Q)}$$

as the instanenous daily death rate.

Now that we have vector control dependent expressions for all relevant parameters, these can be substituted into our equations to calculate key transmission measures, such as R_0. In the presence of vector control measures, we relabel R_0 as the basic reproductive number under control, R_c.

Entomological inoculation rate

Using above expressions for a, m and D, we have:

$$E = maK^N D\frac{1}{M} \quad \text{(R)}$$
$$= \frac{M}{H}\frac{Q}{\delta}K^N B_0 \sum_{i=0}^{n}[K^i - K^i(1-\kappa)^i]\frac{1}{M} \quad \text{(S)}$$

$$= \frac{1}{H} K^N B_0 \sum_{i=0}^{n} [K^i - K^i(1-\kappa)^i] \tag{T}$$

$$\approx \frac{B_0 K^{N+1}[(K-1)(1-\kappa)^{n+1}K^n + (1-K+\kappa K)K^n - \kappa]}{H(K-1)(1-K+\kappa K)} . \tag{U}$$

Vectorial capacity

Using above expressions for a, m and g, we have:

$$V = \frac{ma^2 p^v}{-\ln(p)} = \frac{ma^2}{g} e^{-gv} \tag{V}$$

$$= \frac{M}{H} \frac{a^2}{g} e^{-gv} \tag{W}$$

$$= -\frac{B_0(1-K^{n+1})}{H(1-K)} \frac{Q^2 K^{v/\delta}}{\delta \ln(K)} . \tag{X}$$

Reproductive number under control

Using above expression for V we have that

$$R_c = \frac{Vbc}{r} = -\frac{bc}{r} \frac{B_0(1-K^{n+1})}{H(1-K)} \frac{Q^2 K^{v/\delta}}{\delta \ln(K)} . \tag{Y}$$

References

[1] Ngufor, C., N'Guessan, R., Boko, P., Odjo, A., Vigninou, E., Asidi, A., Akogbeto, M., Rowland, M.: Combining indoor residual spraying with chlorfenapyr and long-lasting insecticidal bed nets for improved control of pyrethroid-resistant anopheles gambiae: an experimental hut trial in benin. Malaria Journal **10**(1), 343 (2011)

[2] Wanjala, C.L., Zhou, G., Mbugi, J., Simbauni, J., Afrane, Y.A., Ototo, E., Gesuge, M., Atieli, H., Githeko, A.K., Yan, G.: Insecticidal decay effects of long-lasting insecticide nets and indoor residual spraying on Anopheles gambiae and Anopheles arabiensis in Western Kenya. Parasites & Vectors **8**(1), 1–10 (2015)

[3] Sherrard-Smith, E., Griffin, J.T., Winskill, P., Corbel, V., Pennetier, C., Djénontin, A., Moore, S., Richardson, J.H., Müller, P., Edi, C., *et al.*: Systematic review of indoor residual spray efficacy and effectiveness against Plasmodium falciparum in Africa. Nature Communications **9**(1), 1–13 (2018)

[4] Kroeger, A., Horstick, O., Riedl, C., Kaiser, A., Becker, N.: The potential for malaria control with the biological larvicide bacillus thuringiensis israelensis (bti) in peru and ecuador. Acta Tropica **60**(1), 47–57 (1995)

[5] Killeen, G., McKenzie, F., Foy, B., Schieffelin, C., Billingsley, P., Beier, J.: A simplified model for predicting malaria entomologic inoculation rates based on entomologic and parasitologic parameters relevant to control. American Journal of Tropical Medicine and Hygiene **62**(5), 535–544 (2000) https://doi.org/10.4269/ajtmh.2000.62.535

[6] Paaijmans, K.P., Thomas, M.B.: The influence of mosquito resting behaviour and associated microclimate for malaria risk. Malaria journal **10**(1), 183 (2011)

[7] Lardeux, F.J., Tejerina, R.H., Quispe, V., Chavez, T.K.: A physiological time analysis of the duration of the gonotrophic cycle of Anopheles pseudopunctipennis and its implications for malaria transmission in Bolivia. Malaria journal **7**(1), 141 (2008)

[8] Smith, D.L., Battle, K.E., Hay, S.I., Barker, C.M., Scott, T.W., McKenzie, F.E.: Ross, macdonald, and a theory for the dynamics and control of mosquito-transmitted pathogens. PLoS pathogens **8**(4) (2012)

[9] C.D.C: CDC: About Malaria – Anopheles Mosquitoes. Available at: https://www.cdc.gov/malaria/about/biology/mosquitoes/ [Accessed: 10th Dec 2019] (2015)

[10] Le Menach, A., Takala, S., McKenzie, F.E., Perisse, A., Harris, A., Flahault, A., Smith, D.L.: An elaborated feeding cycle model for reductions in vectorial capacity of night-biting mosquitoes by insecticide-treated nets. Malaria journal **6**(1), 10 (2007)

[11] Hughes, A., Lissenden, N., Viana, M., Toé, K.H., Ranson, H.: Anopheles gambiae populations from Burkina Faso show minimal delayed mortality after exposure to insecticide-treated nets. Parasites & vectors **13**(1), 17 (2020)

[12] Smith, D., Drakeley, C., Chiyaka, C., Hay, S.: A quantitative analysis of transmission efficiency versus intensity for malaria. Nature Communications **1**, 108 (2010)

[13] Boyd, M., Kitchen, S.: A consideration of the duration of the intrinsic incubation period in vivax malaria in relation to certain factors affecting the parasites. The American Journal of Tropical Medicine and Hygiene **1**(3), 437–444 (1937)

[14] Gary, R., Foster, W.: Effects of available sugar on the reproductive fitness and vectorial capacity of the malaria vector anopheles gambiae (diptera : Culicidae). Journal of Medical Entomology **38**(1), 22–28 (2001) https://doi.org/10.1603/0022-2585-38.1.22
